# From Editorial Board of Special Issue Entitled “Post-Stroke Rehabilitation”

**DOI:** 10.3390/brainsci14080824

**Published:** 2024-08-16

**Authors:** Noureddin Nakhostin Ansari, Gholamreza Hassanzadeh, Ardalan Shariat

**Affiliations:** 1 Department of Physiotherapy, School of Rehabilitation, Tehran University of Medical Sciences, Tehran P.O. Box 14155-6559, Iran; nakhostin@sina.tums.ac.ir; 2 Research Center for War-Affected People, Tehran University of Medical Sciences, Tehran P.O. Box 14155-6559, Iran; 3 Department of Digital Health, School of Medicine, Tehran University of Medical Sciences, Tehran P.O. Box 14618-84513, Iran; hassanzadeh@tums.ac.ir; 4 Department of Anatomy, School of Medicine, Tehran University of Medical Sciences, Tehran P.O. Box 14176-13151, Iran; 5 Department of Neuroscience and Addiction Studies, School of Advanced Technologies in Medicine, Tehran University of Medical Sciences, Tehran P.O. Box 55469-14177, Iran

Diseases affecting the nervous system are diverse. A recent global burden of disease study found that nervous system diseases were the leading cause of disability-adjusted life-years (DALYs) in 2021 [1]. Among the ten conditions with the highest age-standardised DALYs in 2021, stroke was ranked first [1]. Furthermore, it is noteworthy that low- and middle-income countries bear the highest burden of stroke [2,3]. With increasing global DALYs, effective strategies for the prevention, medical treatment, and rehabilitation of nervous system diseases, particularly stroke, are imperative. It is subsequently crucial to swiftly identify and treat stroke patients, especially in remote or rural areas, to minimise subsequent complications. The time taken to intervene in cases of stroke is particularly critical in reducing the risk of long-term disability and mortality [4]. 

This Special Issue presents the latest achievements related to post-stroke rehabilitation though the publication of 11 papers, comprising several types of articles including original studies, reviews, and a brief report written by experts from Iran, the USA, China, Australia, Italy, Spain, Taiwan, the Netherlands, the UK, and Canada.

An editorial written by Nakhostin Ansari et al., entitled “Telestroke: a novel approach for post-stroke rehabilitation”, highlights the importance of telemedicine in the current world of patient’s rehabilitation post stroke (Contribution 1). In a retrospective cohort study entitled “Association of the neutrophil-to-lymphocyte ratio with 90-day functional outcomes in patients with acute ischemic stroke”, Cheng et al. identify the potential factors associated with functional prognosis in acute ischemic stroke (Contribution 2). 

In a paper by Azarnia et al., the authors designed a double-blind, randomised clinical trial with attention on the effectiveness of uni-hemispheric dual-site anodal tDCS on brain metabolic changes in stroke patients (Contribution 3). After this, Cinnera et al., in their paper entitled “Convergent validity of the timed walking tests with functional ambulatory category in subacute stroke”, assess the convergent validity of three different walking tests for patients post stroke (Contribution 4). Then, Chilvers et al. published an interesting paper entitled “Clinical, neuroimaging and robotic measures predict long-term proprioceptive impairments following stroke” with a focus on robotic measures to predict proprioceptive impairments among patients post stroke (Contribution 5).

Authors from Johns Hopkins University, in their paper entitled “Remapping and reconnecting the language network after stroke”, review the literature on neurotypical individuals and individuals with post-stroke aphasia (Contribution 6). Following this, a narrative review entitled “Exploring the prospects of transcranial electrical stimulation (tES) as a therapeutic intervention for post-stroke motor recovery: a narrative review”, conducted by authors from the USA, explores the mechanisms underlying commonly employed tES techniques and evaluates their prospective advantages and challenges in applications in motor recovery after stroke (Contribution 7). Then, Shariat et al., in their scoping review paper entitled “Outcome measures utilized to assess the efficacy of telerehabilitation for post-stroke rehabilitation: a scoping review”, determine the outcome measures used in TR studies and define which parts of the International Organization of Functioning are measured in trials (Contribution 8). Then, Ko et al., from Taiwan, present a systematic review and meta analyses entitled “The application of soft robotic gloves in stroke patients: a systematic review and meta-analysis of randomized controlled trials” and determine the effectiveness of soft robotic gloves (SRGs) in improving the motor recovery and functional abilities in patients with post-stroke hemiparesis (Contribution 9). Moreover, Chacon-Barba et al., from Spain, published their systematic review entitled “Effects of resistance training on spasticity in people with stroke: a systematic review” in which they analyse the effects of resistance training, compared with no treatment, conventional therapy, or other therapies, in people with stroke-related spasticity (Contribution 10). 

Finally, Dadbakhsh et al. conclude this Special Issue with their brief review entitled “Translation, adaptation, and determining the intra-rater reliability of the balance evaluation systems test (BESTest) for Persian patients with chronic stroke” (Contribution 11). 

Overall, this Special Issue highlights the need to focus on multiple variables related to the assessment and rehabilitation of post-stroke patients, utilising not only traditional methods, but also advanced technologies such as telehealth and robotics. 

We believe that continuing research in this field is imperative for conducting more in-depth investigations, particularly concerning cost and feasibility, especially for those living in rural areas who lack access to specialists and hospitals in big cities. In this context, the development of novel protocols and approaches, such as telehealth, should be given significant consideration.
